# Exosome Proteomics of SOD1^D90A^ Mutation Suggest Early Disease Mechanisms, and FN1 as a Biomarker

**DOI:** 10.1002/acn3.70208

**Published:** 2025-09-29

**Authors:** Mukesh Gautam, Ali Laith, Aslihan Gunel, Melda Yilmaz, Nazli Basak, Halil Idrisoglu, P. Hande Ozdinler

**Affiliations:** ^1^ Department of Neurology, Feinberg School of Medicine Northwestern University Chicago Illinois USA; ^2^ Department of Chemistry and Biochemistry Kirsehir Ahi Evran University Kirsehir Turkiye; ^3^ Medical Sciences Department Kocaeli University Izmit Turkiye; ^4^ School of Medicine, KUTTAM‐NDAL Koc University Istanbul Turkiye; ^5^ School of Medicine Department of Neurology, Istanbul University Istanbul Turkiye; ^6^ Feinberg School of Medicine Les Turner ALS Center, Northwestern University Chicago Illinois USA; ^7^ Chemistry of Life Processes Institute Northwestern University Evanston Illinois USA; ^8^ Feinberg School of Medicine Mesulam Center for Cognitive Neurology and Alzheimer's Disease, Northwestern University Chicago Illinois USA

**Keywords:** ALS, biomarker, exosomes, proteomics, SOD1

## Abstract

Amyotrophic lateral sclerosis (ALS) is a neuromuscular disease. Super oxide dismutase 1 (*SOD1*) gene mutations cause ALS, and the *D90A* mutation is associated with primarily upper motor neuron (UMN) loss.

**Objective:**

Our goal is to reveal the early cellular events in ALS pathology and identify potential pharmacokinetic biomarkers, using well‐defined patient populations.

**Methods:**

Exosomes are isolated from serum either single or multiple time points from members of one family, who have SOD1^D90A^ mutation, and their protein content is assessed by tandem mass‐spec proteomics. Ingenuity Pathway analysis is used to highlight cellular events that are perturbed as the disease progressed. The linear regression analysis, using ALSFRS scores of patients and the protein content, helps identify potential pharmacokinetic biomarkers, which are confirmed with the ELISA assay.

**Results:**

Father, Son, and Daughter are at different disease stages and carry the SOD1^D90A^ mutation. Albeit, the Daughter remained asymptomatic within a year; she had significant biological changes. The Son transitioned from asymptomatic to early symptomatic within a year, while the Father was symptomatic. Patient #2, who also had the SOD1^D90A^ mutation, was more advanced. Comparison of the Son, Father, and Patient #2 suggested Fibronectin1 (FN1) as a potential pharmacokinetic biomarker, which is confirmed by ELISA.

**Interpretation:**

Exosome proteomics offer a powerful approach to interrogate disease‐specific or disease‐related proteins that become present in the blood. This helps define the perturbed cellular events with respect to disease progression and reveal potential pharmacokinetic biomarkers. We find FN1 levels to increase with disease progression, suggesting it may be a pharmacokinetic biomarker, especially for ALS patients with prominent UMN loss.

## 1 Introduction

Amyotrophic lateral sclerosis (ALS) is a complex and heterogeneous disease, affecting both the cortical and the spinal components of motor neuron circuitry [1, 2]. It can be classified as familial and sporadic, based on its pattern of inheritance. In familial ALS, the disease‐causing mutations are carried from generation to generation. Today, more than 150 genes are either linked or associated with ALS pathology [3, 4]. Superoxide dismutase (SOD1) was one of the first genes discovered causing ALS, when mutated [5, 6]. More than 150 mutations have been identified in this gene [7, 8]. Interestingly, the *D90A* mutation is primarily detected in ALS patients with prominent upper motor neuron (UMN) loss [9].

Lack of SOD1 protein is not lethal, but the presence of the mutated version introduces “gain of toxicity,” suggesting that removal of the mutated protein would be therapeutic [10, 11]. Therefore, anti‐sense oligonucleotides (ASOs) offer a great potential for effective treatment strategies, especially for familial ALS patients with known mutations [12, 13]. Qalsody was developed using ASO technology, especially for patients with SOD1 mutations [14]. Currently, Qalsody has been approved in the United States, Europe, and in Japan; it is becoming broadly available to patients with SOD1 mutations around the globe. Therefore, it is important to understand the early stages of the disease and develop novel ways to measure disease progression or improvement in patients.

**FIGURE 1:**
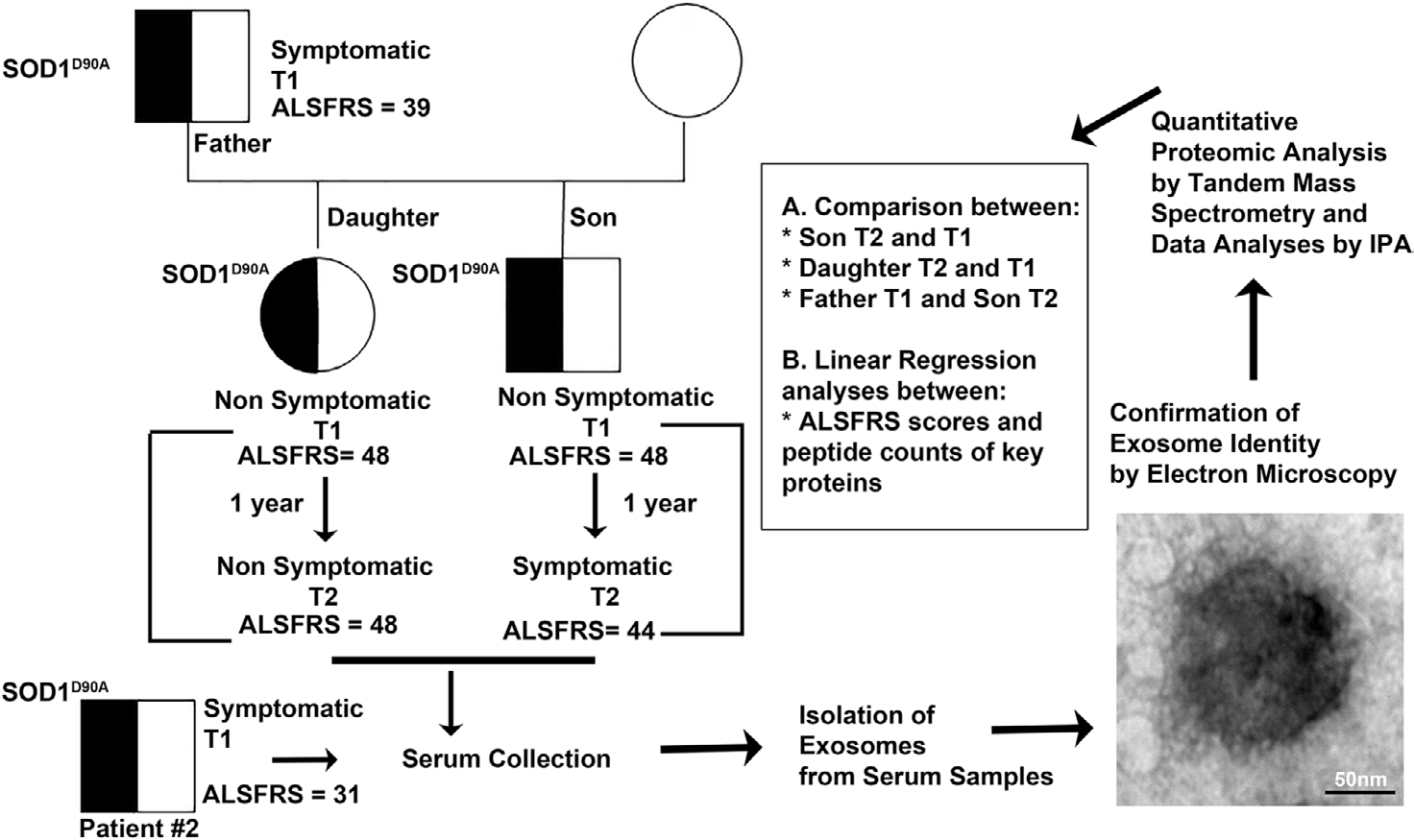
Schematic representation for the choice of family and study design. A family with SOD1^D90A^ mutation was included in the case study. The Father was symptomatic at the time of study. Serum was collected from Daughter and Son at two time points, separated by 1 year. The Daughter remained asymptomatic at both T1 and T2, whereas the Son progressed from asymptomatic (T1) to symptomatic (T2). Patient #2, who has the same SOD1^D90A^ mutation and is at a more progressed disease stage was also included in the study. Exosomes were isolated from the serum samples and their exosomal identity was confirmed by electron microscopy before being utilized for quantitative proteomics analysis by Tandem Mass Spectrometry. Quantitative changes in the spectral counts of peptides and proteins were analyzed by large‐data management toolboxes to investigate altered canonical pathways, changes in cellular functions and disease‐related network analyses.

We had access to the blood samples of patients with the same mutation, in the same family, but at different stages of their disease and with decreasing amyotrophic lateral sclerosis functional rating scale (ALSFRS) scores. The Father had been symptomatic for 2 years, and he gave blood only at one time point. Interestingly, the Daughter remained asymptomatic, but the Son transitioned from asymptomatic to symptomatic stage within a year. We took advantage of the exosomes present in their serum and utilized a tandem mass‐spec based proteomic approach to assess the protein content in all affected family members, with high precision, and studied the changes in the protein content over time (Figure 1).

Exosomes are small vesicles packed with proteins and biomolecules [15, 16]. Their content is strictly controlled as they are used to send signals across tissues and to distant locations within the body [17, 18]. We reasoned that their content would be informative about disease state. We performed bottom‐up proteomics and investigated identified proteins with the help of large‐data management toolboxes, such as ingenuity pathway analyses (IPA), which highlight canonical pathways, upstream regulators, and disease mechanisms that are most relevant. Exosome proteomics helped us understand the changes that occurred in the Son as he became symptomatic and in the Daughter, even though she remained asymptomatic. More importantly, linear regression analyses using ALSFRS scores and the concentration of proteins highlighted the presence of key proteins that may be informative about the timing and the extent of the disease progression. This could be critically important for the development of pharmacokinetic biomarkers so that disease progression in the patients can be better monitored.

Our study not only reveals the cellular and even molecular basis of the dynamic changes that occur in patients prior to symptom onset and as the disease progresses, it also suggests a potential utility for Fibronectin1 (FN1) as a potential pharmacokinetic biomarker, especially for ALS patients with SOD1^D90A^ mutation and ALS patients with prominent UMN loss.

## 2 Materials and Methods

### 2.1 Patient Selection

The Father (male; age = 56, ALSFRS = 39), Son (male; T1: age = 30, ALSFRS = 48; T2: age = 31, ALSFRS = 44), and Daughter (female; T1: age = 24, ALSFRS = 48, T2: age = 25, ALSFRS = 48) had *D90A* mutations in their *SOD1* gene. Patient #2, who had the same *D90A* mutation, was more advanced in the disease (male; age = 56; ALSFRS = 31). Both Patient #2 and the Father were Turkish and had comparable ethnicity. A limited number of sex and age‐matched control cases (*n* = 2 male, *n* = 1 female) were originally included in the study, however, the extent of variation among them did not enable them to be used as a “control group.” Therefore, comparisons were made within time points of each patient, using their T1 as baseline.

For ELISA, Patient #3: (male with TDP‐43^G348C^ mutation; T1: age = 38, ALSFRS = 40; T2: age = 39, ALSFRS = 36), and Patient #4: (male with PABPN1^Ala11dub^ intron expansion mutation ((NM_004643.3) c.21_23dup(p.Ala11dup)); T1: age = 26, ALSFRS = 37; T2: age = 26, ALSFRS = 32) were included. Both patients had prominent UMN involvement. Control #1: (male, age = 37; control for Patient #3) and Control #2: (male, age = 30; control for Patient #4) were used as their age and sex‐matched controls for the ELISA assays.

Exome sequencing was performed to determine mutation(s) in patients, using the Illumina NovaSeq 6000 platform (Macrogen, Seoul, Korea). Bioinformatic evaluation of the raw data was conducted using the SEQ platform (Genomize, Istanbul, Turkey), aligning the reads to the hg19 reference assembly. Non‐coding and synonymous variants other than those of splice regions were filtered out. Analysis of all rare variants (MAF < 0.01) in genes registered in the OMIM database was used, and results for each patient were confirmed with Sanger sequencing.

All blood samples were collected with an approved IRB (PI: Dr. Aslihan Gunel; #: 2017‐19/228; Date = 12/12/20‐17; Kirsehir Ahievran University, Medical School, Kirsehir/Turkiye). Patient's ALSFRS scores were determined by Dr. Halil Idrisoglu MD at the time of blood collection. The same ALSFRS scale, that is applied universally and measures 12 different aspects of physical function [19, 20, 21], was utilized. Each participant signed the consent form prior to inclusion in the study.

### 2.2 Serum Isolation

Blood is isolated from Son and Daughter at two time points (T1 and T2; separated by 1 year). Blood is collected from Father and Patient #2 only at one time point. Whole blood (5 mL) was collected on red‐topped Vacutainer tubes (Becton Dickinson), allowed to coagulate at room temperature (RT) for 1 h, centrifuged at 1000–2000*g* for 10 min at 4°C, and the serum was collected.

### 2.3 Isolation and Confirmation of Exosomes

Exosomes from the serum were isolated using the ExoEasy kit (Qiagen). Briefly, 200 μL serum was mixed with 800 μL PBS and filtered through a 0.4 mm polypropylene filter (Whatman). The filtrate was loaded onto an ExoEasy maxi‐spin column and centrifuged (500 g for 10 min). The flowthrough was discarded and the column was treated with buffer XB for 5 min before centrifuging (5000*g* for 5 min). The column was eluted with buffer E, and the eluted exosomes were further ultracentrifuged (100,000*g* for 90 min; Beckman Coulter LE‐80). A fraction of the exosome pellet was used for electron microscopic confirmation of exosome identity, and the rest of the exosome pellet was used for protein extraction.

EM analysis is one of the most appropriate approach to confirm exosome identity, based on size (50–150 nm in diameter; Figure 1). A 10 μL drop of exosome‐enriched solution was put on carbon–formvar coated lead mesh grids for 1 h. Samples were washed with PBS, post‐fixed in 2% paraformaldehyde and 2.5% glutaraldehyde for 10 min. The grids were washed with deionized water and samples were contrasted by incubating in 2% uranyl acetate followed by embedding in 0.13% methyl cellulose and 0.04% uranyl acetate. The grids were dried and visualized using FEI Tecnai Spirit Transmission Electron Microscope at 120 kV. Images were captured on FEI Eagle camera using FEI TIA software.

### 2.4 Protein Extraction From Exosome and Proteomics

Protein was extracted from exosomes using the methanol–chloroform method [22]. Briefly, exosome pellets were suspended in 100 μL RIPA buffer with a protease inhibitor, 400 μL methanol was added, and the pellet was vortexed. Quantitative proteomic analysis was performed on exosomes as previously reported [23]. Briefly, the exosome pellet was lysed in RIPA buffer, proteins were precipitated using chloroform/methanol, were denatured in 8 M urea with surfactant, and digested overnight with 2 μg of trypsin at 37°C. Peptide concentration was measured and loaded into Ultra Performance Liquid Chromatography (UPLC) to ensure equal amounts of material are assayed. Peptides are electro‐sprayed and analyzed by high‐resolution tandem MS, as previously explained [23, 24], and a detailed protocol can be found at File S1. The raw data are extracted and searched against the species‐matched protein database. Sequest/Prolucid, Mascot, X! Tandem, and OMSSA are used to achieve confident protein identifications [25, 26]. This dataset is highly confident at a protein level with a 1% false detection rate (FDR).

A peptide confidence of 0.95 was set as the minimum threshold. The false discovery rate (FDR) is calculated as the percentage of reverse decoy PSMs among all the PSMs that passed the confidence threshold. Each protein identified is required to have a minimum of one half‐tryptic peptide; however, this peptide had to be an excellent match with an FDR less than 0.001.

### 2.5 Comparative Analysis of Proteins

The common proteins that are present in the exosomes of patients' serum were identified using Sequest/Prolucid, Mascot, X! Tandem, and OMSSA, all of which can achieve confident protein identifications. This protocol requires some label‐free semi‐quantitative measure of abundance to be provided from the analysis software, which could be in the form of spectral counts and ion intensities.

The biological functions and cellular events were investigated using Ingenuity Pathway analysis (IPA), with the following settings: Relationship to consider = direct relationships; node types = all; data source = all; confidence = only experimentally observed; species = mammal; and filter = stringent filter. The canonical pathway graphs were sorted with respect to *z*‐score, a statistical measurement of the score's relationship to the mean in a group. The positive *z*‐scores mean the change is above the mean (increase). IPA calculates *p* values using the right‐tailed Fisher's Exact Test. *p* < 0.005 was considered significant. Because the 3 controls that were originally chosen for Brother, Father, and the Daughter had too much internal variation, they could not serve as a coherent “control” group and did not allow any feasible comparative analyses. Therefore, T1 point was used as a baseline and individual control for the T2 time point for the same patient.

Linear regression analysis was performed to investigate the presence of a potential correlation between the ALSFRS scores (independent variable) and the quantitative assessment of spectral counts of peptides from distinct proteins (dependent variable). The coefficient of determination, the “*R* squared” is determined for each protein and its isoform. Prism 10 software was used to perform simple linear regression using ALSFRS scores of the daughter, the son, father, and Patient #2, as well as the quantitative values of the spectral counts for the peptides and the proteins of interest.

### 2.6 ELISA Confirmation

ELISA was used to quantify levels of FN1 protein in the serum (Cat. # DFBN10, R&D Systems). Briefly, 10 μL serum was diluted 10,000 times with RD5P solution. Serial dilution of human FN1 standards was prepared to make the standard curve. Samples and standards were added to a pre‐coated 96‐well ELISA plate with 100 μL RD1‐9 assay diluent and incubated at RT for 2 h. Wells were washed with wash buffer and incubated with 200 μL/well Human Fibronectin conjugate at RT for 2 h. Wells were washed, 200 μL/well substrate solution was added, and incubated at RT for 30 mins. 50 μL stop solution was added, and optical density was determined at 450 nm using an ELISA plate reader (BioTek). Wavelength correction was performed at 540 nm. The concentration of FN1 was calculated using the standard curve.

## 3 Results

This study was made possible by members of an ALS family, carrying the SOD1^D90A^ mutation, and Patient #2—not from the same family, but from the same ethnicity—who had the same mutation and was further along in the disease (Figure 1). The family had a Daughter and a Son; both were asymptomatic (ALSFRS = 48) at the time of first blood collection (T1). After 1 year (T2), the Son became symptomatic (ALSFRS = 44), while the Daughter remained asymptomatic (ALSFRS = 48). The Father was symptomatic (ALSFRS = 39) at the time of blood collection. Patient #2 represents a disease stage that is more advanced than the Father (ALSFRS = 31). The Daughter, Son, and Father, with the same mutations but at different stages of their disease, allowed an unprecedented opportunity to assess the cellular underpinnings of disease progression. Inclusion of Patient #2 allowed linear regression analyses to be performed to further assess a potential correlation with ALSFRS scores and the levels of key proteins.

The proteomic experiments allow assessment of spectral counts for peptides that correspond to given proteins and their isoforms, such that quantitation can be achieved even at femtogram‐level precision [23]. Thanks to unbiased and sensitive quantitation, we were able to study changes over time.

We utilized IPA to study the list of proteins that change and what that means at a cellular level. Four key analyses were performed: canonical pathway, diseases and functions, upstream regulators, and networks. The canonical pathway analyses revealed how identified proteins are distributed among the established canonical pathways and whether this distribution can be explained by luck or not. Having a significant *p* value, high coverage ratio, and a positive *z*‐score with suggested bias of activation helped reveal cellular events that are positively associated with the identified proteins. In addition, the potential upstream regulators and protein–protein interaction networks highlight key cellular events as well as the proteins associated with them. These unbiased large‐data management toolboxes, combined with curated findings and artificial intelligence, offer great advantages to highlight key cellular events and proteins of interest that are likely associated with disease progression for each patient.

### 3.1 The Cellular Changes That Occur in the Daughter

**FIGURE 2:**
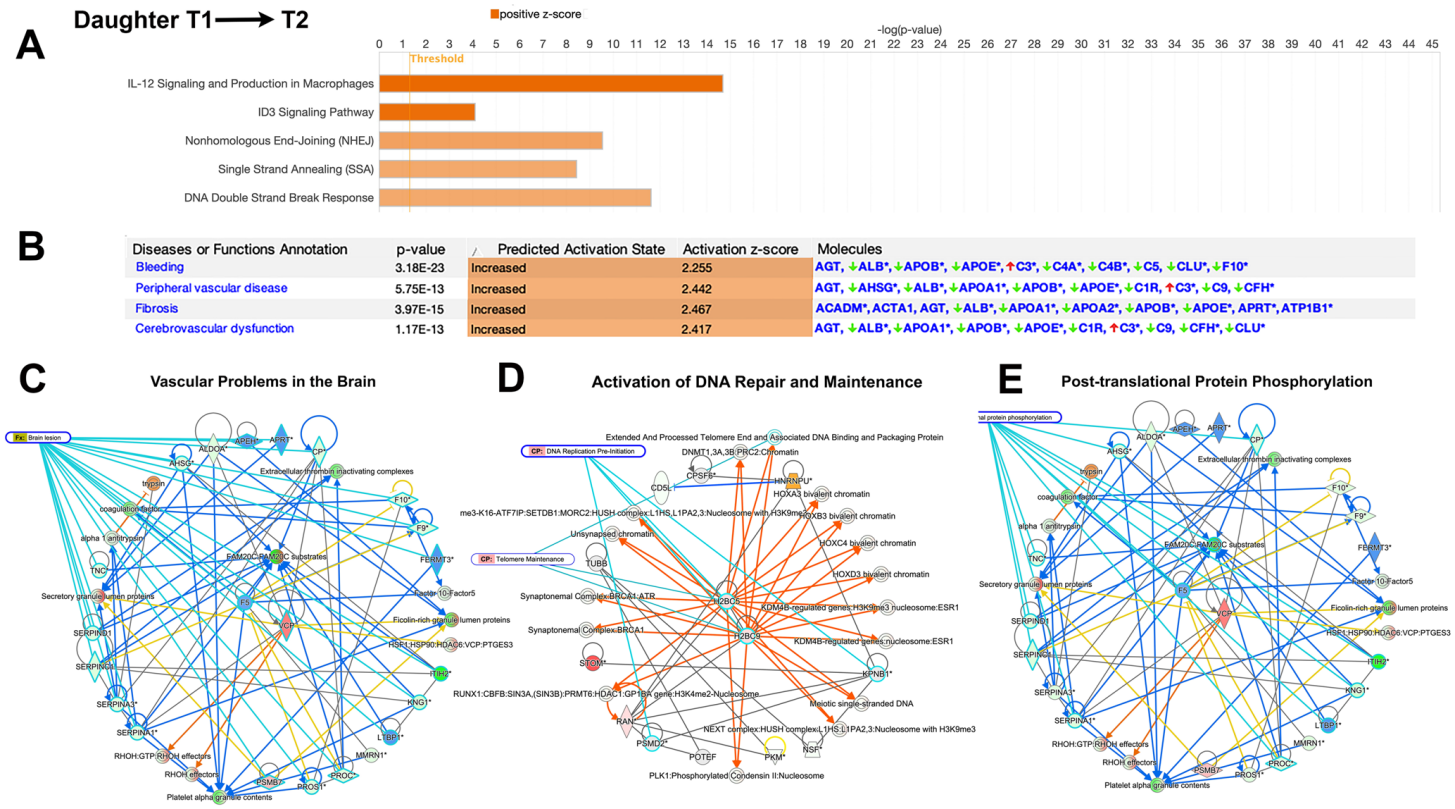
Analysis of proteins that differ between T1 and T2 time points of the Daughter who remained asymptomatic. (A) Canonical pathway analyses of proteins that display differences between T1 and T2 time points of the Daughter. Orange depicts a positive *z*‐score (increased relevance). (B) Analyses of diseases and cellular functions which have the highest coverage among the given proteins. First column depicts the cellular event, second column shows the *p* value, the predicted activation state together with the activation *z*‐score suggests the involvement of the cellular event, and the last column shows some of the most prominent proteins involved in each cellular event. Network analyses highlighted three important cellular events that take place during this time; Vascular Problems in the Brain (C), Activation of Brain Repair and Maintenance pathways (D), Post‐translational protein phosphorylation and (E).

Even though the Daughter remained asymptomatic (ALSFRS = 48 at both T1 and T2), striking differences were noted. A total of 211 proteins were different, while some increased (*n* = 53) and most decreased (*n* = 158; Table S1). When these proteins were investigated by IPA, three important cellular events were highlighted: (1) problems with maintaining the stability of the blood‐brain barrier (BBB); (2) effort to maintain the stability of the genome; (3) initiation of the first steps of the neuroimmune reaction. IL‐12 signaling and production in macrophages (ratio = 21/242; *p* = 3.79E‐16; z = 3.3), and the ID3 signaling pathway (ratio = 24/967; *p* = 5.94E‐7; *z* = 4.123), both of which are important cellular events that represent immediate‐early responses to mitogenic signals and oxidative stress [27, 28, 29], suggested an early stage of neuroimmune modulation, even though she remained asymptomatic (Figure 2A). Nonhomologous end joining (ratio = 9/49; *p* = 1.38E‐10; *z* = 1.89), single‐strand annealing (ratio = 8/114; *p* = 3.19E‐6; *z* = 1.89), and DNA double‐strand break response (ratio = 8/59; *p* = 1.85E‐8; *z* = 1.890) were significantly upregulated with a positive z‐score, revealing her body's effort in maintaining genomic stability.

The disease and functions annotations suggested presence of a peripheral vascular disease (*p* = 5.75E‐13; *z* = 2.402), bleeding (*p* = 3.18E‐13; *z* = 2.288), and cardiovascular dysfunction (*p* = 1.17E‐13; *z* = 2.417), all pointing to major problems with the stability and integrity of blood vessels at this very early non‐symptomatic stage of the disease (Figure 2B).

Network analyses of 211 proteins revealed how these proteins interacted with each other and what this interaction means in terms of cellular activities. Vascular problems in the brain (Figure 2C) were selected as one of the key cellular events (*n* = 17 proteins). Activation of DNA repair and maintenance was also highlighted (*n* = 8 proteins) (Figure 2D). An association with an increase in post‐translational protein phosphorylation was also noted (Figure 2E). Interestingly, VCP protein, which is linked to ALS when mutated [30] was one of the key interactomes of the network. A second network suggested the relevance of DNA repair with proteins that are important for telomerase repair and the DNA replication pre‐initiation pathway. The Daughter, albeit asymptomatic, seemed to be having major vasculature problems, increased protein phosphorylation, and that she was trying to maintain genomic integrity and cellular homeostasis at this stage while initiating the first phases of the neuroimmune reaction.

### 3.2 The Cellular Changes That Occur in the Son

**FIGURE 3:**
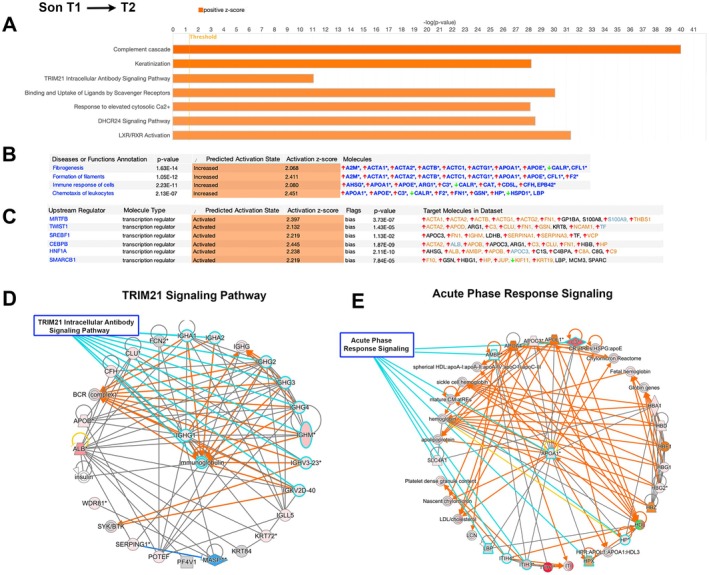
Analysis of proteins that differ between T1 and T2 time points of the Son who transitioned from asymptomatic to symptomatic stage. (A) Canonical pathway analyses of proteins that display differences between T1 and T2 time points of the Son. Orange depicts a positive *z*‐score (increased relevance) (B) Analyses of diseases and cellular functions which have the highest coverage among the given proteins. First column depicts the cellular event, second column shows the *p* value, the predicted activation state together with the activation z‐score suggests the involvement of the cellular event, and the last column shows some of the most prominent proteins involved in each cellular event. (C) Table of upstream transcriptional regulators that are marked as “biased,” with predicted activation state, *z*‐score, and significant *p* value. The last column includes examples of genes that are under the control of the given transcriptional regulator. Network analysis highlight the presence of TRIM21 Signaling Pathway (D) and Acute Phase Response Signaling Pathway (E).

The Son became symptomatic within the same year. When the proteomic results obtained from exosomes isolated at T1 and T2 were compared, 275 proteins were identified to be either increasing (*n* = 205) or decreasing (*n* = 70; Table S2). The canonical pathway analyses suggested a robust upregulation of complement cascade (ratio = 36/136; *p* = 1.02E‐40; *z* = 5.196), keratinization (ratio = 33/216; *p* = 6.05E‐29; *z* = 4.690), TRIM21 signaling pathway (ratio = 28/567; *p* = 8.19E‐12; *z* = 4.243), response to elevated cytosolic Ca^+2^ (ratio = 28/132; *p* = 7.01E‐29; *z* = 4.025), DHCR24 signaling pathway (ratio = 29/143; *p* = 2.85E‐29; *z* = 3.9), and LXR/RXR activation (ratio = 30/130; *p* = 4.54E‐32; *z* = 3.9) (Figure 3A).

The diseases and functions annotations suggested an increase in two important cellular events: one is an increase in the formation of filaments (*p* = 1.63E‐14; *z* = 2.068) and fibrogenesis (*p* = 1.05E‐12; *z* = 2.411), and the other is an increase in the immune response of cells (*p* = 2.23E‐11; *z* = 2.080), with an increase in the chemotaxis of leukocytes (*p* = 2.451; *z* = 2.451).

IPA also suggested an increase in gene expression with the presence of transcription factors that may be upregulated, with *p* values indicating significance and positive z‐scores. Transcriptional regulators, such as CCAAT Enhancer Binding Protein Beta (CEBPB), which regulates the expression of genes involved in immune and inflammatory responses [31, 32, 33, 34], were suggested to be activated during this time (Figure 3C).

The network analyses of these 275 proteins suggested the interaction of key proteins and how this may help explain cellular events that are altered. For example, activation of the TRIM21 signaling pathway (*n* = 10 proteins; Figure 3D) and acute phase response signaling (*n* = 8 proteins; Figure 3E) were noted with the involvement and interaction of numerous proteins within a functional network. The proteins detected in the daughter and the son were strikingly different, and the cellular events that were highlighted by IPA also suggested significant differences. As the Son became symptomatic, he had a dramatic increase in his immune response, and activation of the TRIM21 signaling pathway also suggested that he suffered problems with mitochondrial integrity as one of the underlying causes of enhanced immune reaction [35, 36, 37].

### 3.3 From Son to Father

The Father represents a more advanced time point than T2 time point of the Son (Father ALSFRS = 39; T2 Son ALSFRS = 44). While the Son just began to show symptoms at T2, the Father had been displaying characteristics of the disease for the past 2 years. We reasoned that the father represents what the Son could be displaying in the future, and therefore compared the results of the Father's exosome proteomic results with that of the T2 time point of the Son. There were 221 proteins that were different between them, some increasing (*n* = 61) and most decreasing (*n* = 160; Table S3).

**FIGURE 4:**
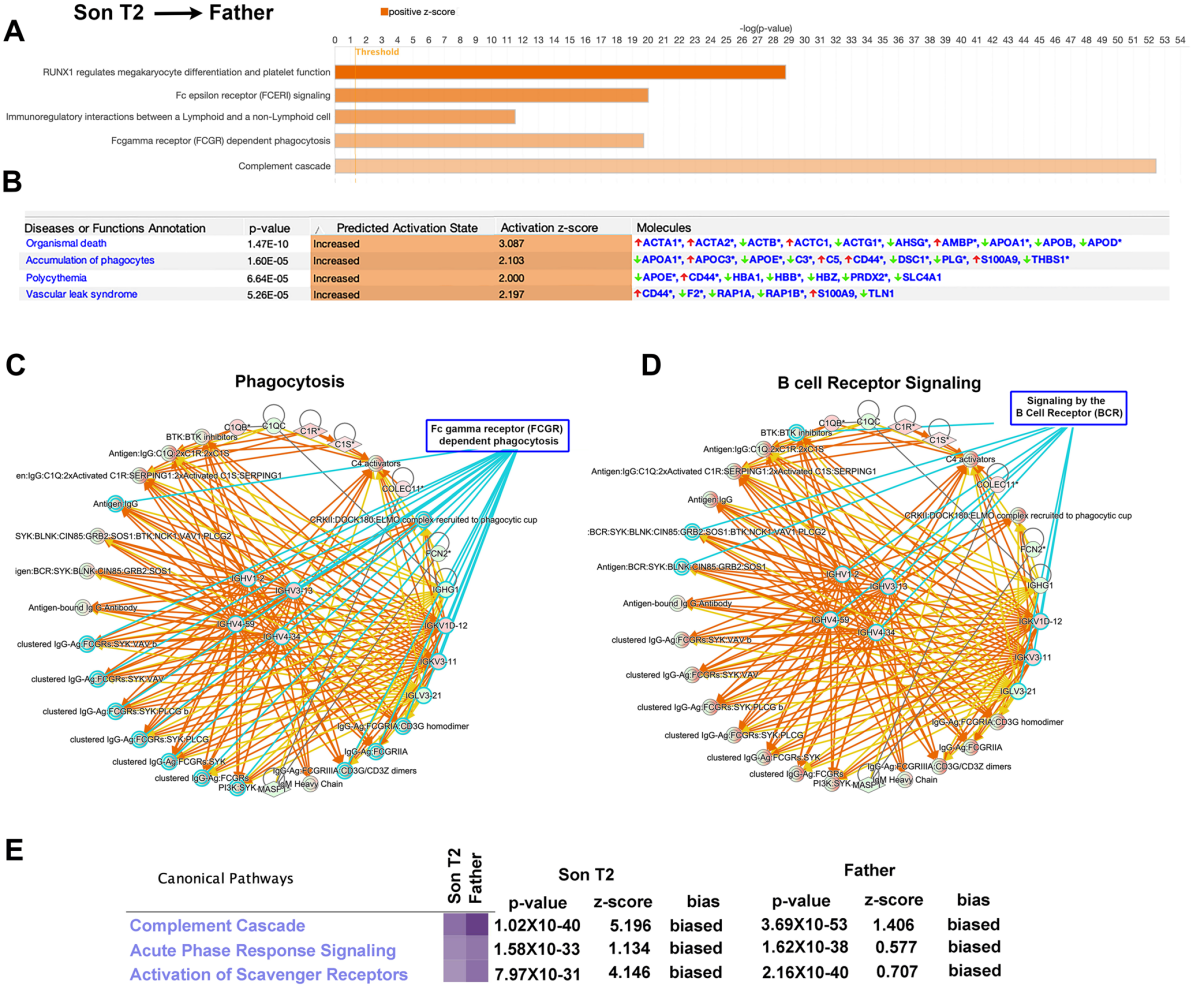
Analyses of proteins that differ between T2 time point of the Son, when he first becomes symptomatic and the Father, who had been symptomatic for 2 years. (A) Canonical pathway analyses of proteins that display differences between T2 time point of the Son and the Father. Orange depicts a positive *z*‐score (increased relevance). (B) Analyses of diseases and cellular functions which have the highest coverage among the given proteins. First column depicts the cellular event, second column shows the *p* value, the predicted activation state together with the activation *z*‐score suggests the involvement of the cellular event, and the last column shows some of the most prominent proteins involved in each cellular event. Network analyses highlight activation of B‐cell receptor signaling (C) and Phagocytosis (D). (E) Comparative canonical pathway analyses between T2 time point of the Son and the Father highlight cellular events that become increasingly significant and relevant with disease progression.

This time, in addition to complement cascade activation (ratio: 41/136; *p* = 3.69E‐53; *z* = 1.406), more detailed aspects of the immune reaction became apparent (Figure 4A). For example, RUNX1 mediated megakaryocyte differentiation (ratio: 21/58; *p* = 1.62E‐29; *z* = 3.441), Fc epsilon receptor signaling (ratio: 24/206; *p* = 9.64E‐21; *z* = 2.5), immunoregulatory interactions between lymphoid and non‐lymphoid cells (ratio: 17/215; *p* = 3.07E‐12; *z* = 2.183), and Fc gamma receptor mediated phagocytosis (ratio: 22/164; *p* = 1.85E‐20; *z* = 1.706) were highlighted to be primarily involved, with high significance that cannot be explained by luck.

Diseases and Function Annotation (Figure 4B) suggested an increase in the accumulation of phagocytes (*p* = 1.6E‐5; *z* = 2.103) and organismal death (*p* = 1.47E‐10; *z* = 3.087). Vascular leak syndrome (*p* = 5.26E‐5; *z* = 2.197) and signs of polycythemia (*p* = 6.64E‐5; *z* = 2.00), which indicate increased red blood cells in the vasculature, were also suggested to be upregulated, further suggesting a continuation of problems that are related to the stability of blood vessels and activation of the cell‐death pathways.

The network analyses were more revealing. There has been an immense upregulation of immune reactions, especially B‐cell receptor signaling (*n* = 10 proteins, Figure 4C), and phagocytosis (*n* = 20 proteins; Figure 4D) emerged for the first time. The most prominent finding was on the significant increase in all aspects of immune reactions. At this stage, the DNA repair mechanisms or cellular events that are crucial to maintaining homeostasis were not highlighted. Alas, cellular destruction, death, and phagocytosis were more prominent.

To further assess the presence of canonical pathways that gradually became more significant and relevant as the disease progressed from Son to Father, we performed comparative pathway analyses using the dataset of Son (T2) and Father. The complement cascade of events, acute phase response signaling, and activation of scavenger receptors were highlighted with significant *p* values and *z*‐scores that suggested “bias” and that these associations cannot be explained by sheer luck (Figure 4E).

### 3.4 Identification of FN1

Now that we begin to reveal the dynamic changes that occur with respect to disease progression, we wanted to investigate the presence of a potential pharmacokinetic biomarker, which may inform about the timing and the extent of the disease progression. We focused our attention on the T2 time point of the Son, the data point of the Father, and Patient#2, as these three samples represented patients at 3 different disease stages with reducing ALSFRS scores.

We excluded data from the Daughter for two reasons: first, the sex difference, and the second is the incomplete penetrance of the D90A mutation [38], which may suggest that the Daughter has a chance to never develop the disease even though she has the mutation. We also excluded the T1 of the Son because, at the time, he was asymptomatic. Therefore, we included data only from symptomatic patients at different stages of their disease.

**FIGURE 5:**
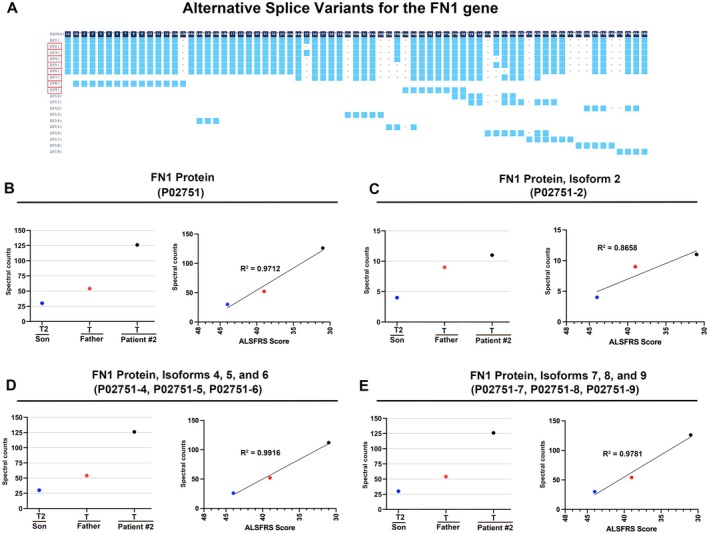
Fibronectin protein becomes increasingly abundant in the exosomes of patients after symptom onset. (A) Schematic representation of Fibronectin (FN1) gene and its isoforms, which are the result of alternative splicing. The isoforms detected in this study are labeled with red box. (B) The FN1 protein levels (as detected by peptide ID P02751) in the Son (T2), Father, and Patient #2. Regression analyses of protein levels with respect to ALSFRS scores. *R* ^2^ = 0.9712. (C) Isoform 2 of FN1 protein levels (as detected by peptide ID P02751‐2) in the Son (T2), Father, and Patient #2. Regression analyses of protein levels with respect to ALSFRS scores. *R* ^2^ = 0.8658. (D) Isoform 4, 5, and 6 of FN1 protein levels (as detected by peptide ID P02751‐4. P02751‐5, P02751‐6, respectively) in the Son (T2), Father, and Patient #2. Regression analyses of protein levels with respect to ALSFRS scores. *R* ^2^ = 0.9916. (E) Isoform 7, 8, and 9 of FN1 protein levels (as detected by peptide ID P02751‐7, P02751‐8, P02751‐9, respectively) in the Son (T2), Father, and Patient #2. Regression analyses of protein levels with respect to ALSFRS scores. *R* ^2^ = 0.9712. Blue dot = Son; red dot = Father; black dot = Patient #2.

In search of proteins that display progressive change among these 3 data points (Son, Father, Patient#2), we found Fibronectin1 (FN1) showing a gradual increase with disease progression. No protein with a gradual decrease was noted. FN1 has numerous isoforms [39, 40] (Figure 5), which have not been well‐characterized for their expression profile, but the emerging evidence suggests their involvement in neurodegeneration [41, 42]. FN1, and 8 of its isoforms, namely isoforms 2, 4, 5, 6, 7, 8, and 9, also displayed a gradual increase (Figure 5B–E).

A strong linear correlation was observed with the levels of FN1 protein and the ALSFRS scores, such that as the disease progressed with reduced ALSFRS scores, the levels of the FN1 protein were increasing in the exosomes isolated from the serum of patients (FN1: Peptide ID = P02751; *R* ^2^ = 0.9712, Figure 5B). Similar results were observed with the isoforms of FN1, such as isoforms 4, 5, and 6 (Peptide IDs = P02751‐4, P02751‐5, P02751‐6; *R* ^2^ = 0.9916, Figure 5D), and isoforms 7, 8, and 9 (Peptide IDs = P02751‐7, P02751‐8, P02751‐9; *R* ^2^ = 0.9781, Figure 5E). Isoform 2 showed the lowest levels of correlation (Peptide ID = P02751‐2; *R* ^2^ = 0.8658, Figure 5C), still prominent. Another correlation with neurofilament light or heavy chain levels would be preferable. However, neurofilament light or heavy chains were not present in the exosomes isolated from these patients (Table S1), and these proteins were not analyzed at the time of blood collection.

**FIGURE 6:**
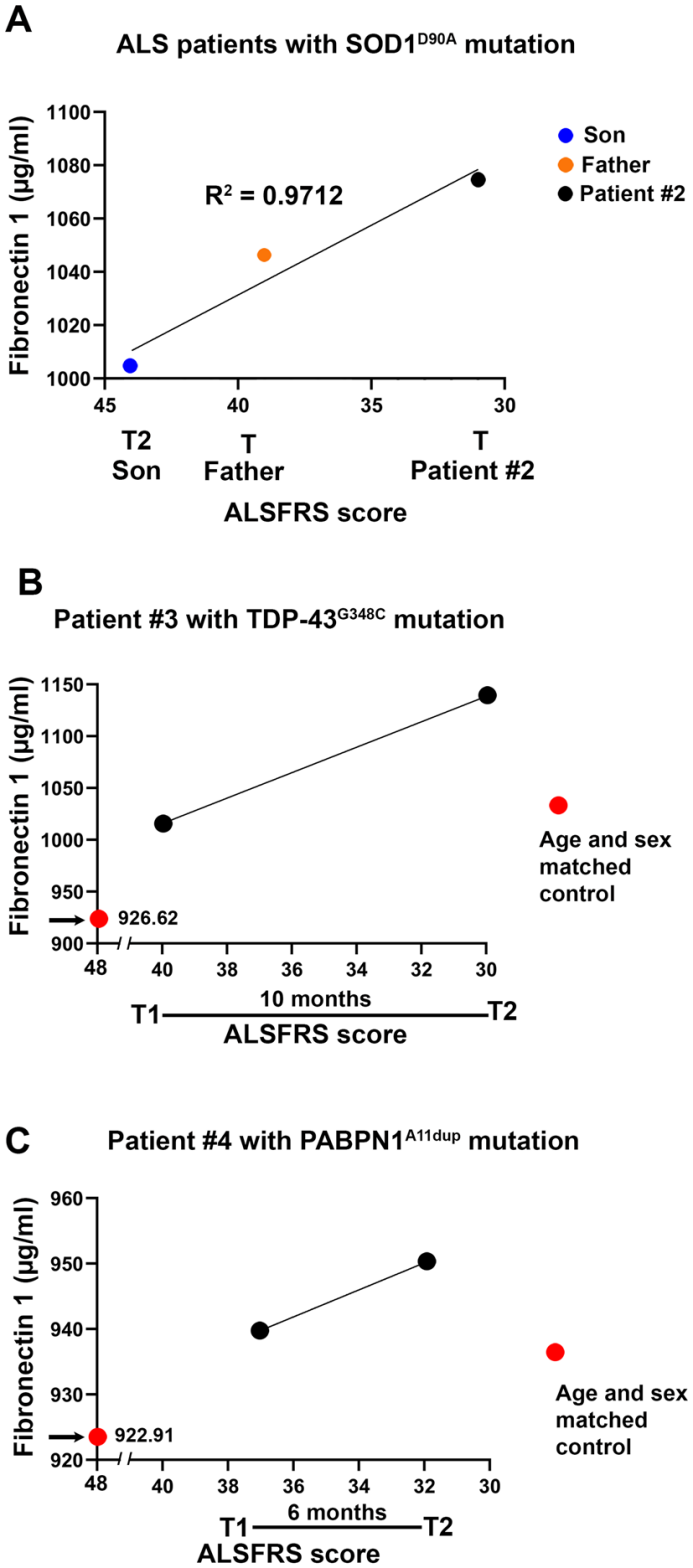
ELISA confirmation of FN1 levels in the serum. (A) FN1 protein was also detected in the serum of the Son, Father and Patient #2, with increasing concentrations. (B) FN1 protein was increasingly present in the serum of an ALS patient, who had TDP‐43^G348C^ mutation (Time between T1 and T2 = 10 months). (C) FN1 protein was increasingly present in the serum of an ALS patient, with PABPN1^Ala11dub^ intron expansion mutation (Time between T1 and T2 = 6 months). Red dot depicts the age and sex‐matched control for each patient.

ELISA assays of serum samples isolated from the Son, Father, and Patient #2 also confirmed the presence of FN1 in the serum and that the levels of protein increased with respect to decreasing ALSFRS scores with a similar correlation (*R* ^2^ = 0.9712; Figure 6A).

We next asked whether FN1 levels would increase only in patients with SOD1^D90A^ mutations, or also in other ALS patients with different mutations. We thus included 2 more patients in the study: Patient #3: (with TDP‐43^G348C^ mutation; T1: age = 38, ALSFRS = 40; T2: age = 39; ALSFRS = 36; time between T1 and T2 = 10 months) Patient #4: (with PABPN1^Ala11dub^, an intron expansion mutation; T1: age = 27, ALSFRS = 37; T2: age = 27, ALSFRS = 32; time between T1 and T2 = 6 months). Both TDP‐43^G348C^ mutation [43, 44] and PABPN1^Ala11dub^ intron expansion mutation [45] have been reported in ALS patients. Both patients were clinically evaluated to be ALS patients with UMN involvement. The FN1 levels in patients were higher than the FN1 levels in their age and sex‐matched controls (red dots). FN1 levels showed a progressive increase in the serum of these 2 unrelated ALS patients with different mutations as their disease progressed (Figure 6B,C).

## 4 Discussion

Our results, using exosomes isolated from the serum samples of well‐defined patients with SOD1^D90A^ mutation, began to suggest the presence of dynamic cellular events that occur at early stages of the disease and highlighted FN1 as a potential pharmacokinetic biomarker, especially for patients with prominent UMN involvement.

ALS is a rare and complex neurodegenerative disorder [46], and developing effective treatment strategies is a major challenge. Neurodegeneration is an extensive process, and clinical symptoms develop much later than neuronal vulnerability and cellular degeneration. It may be possible to detect and track neurodegeneration using patients' biomaterial, such as serum and plasma. These biofluids are rich in proteins and metabolites, which may be secreted by many different cells and organs. Therefore, identification of disease‐relevant biomolecules remains a challenge.

Exosomes come into the picture at this juncture. Exosomes are small extracellular vesicles (50–150 nm) that play crucial roles in molecular trafficking and intercellular transport, facilitating long‐distance communication [47, 48, 49]. Their content has significant information about disease state is tightly regulated [15, 17], and subject to change with respect to disease progression. Because brain‐derived exosomes cross the BBB and enter the blood stream, they provide an accessible window to assess and monitor the alterations of cellular processes in the brain, with respect to disease [50]. This can lay the foundation for improving biomarker discovery for the diagnosis, prognosis, and stratification of patients. We thus studied the protein content of exosomes that are present in the serum of patients who are at different stages of their disease. We find that the BBB is compromised, mitochondrial damage becomes significant, protein aggregations begin to occur, and the immune response is initiated even at the presymptomatic stage.

Utilization of exosomes for biomarker discovery is an emerging field [51, 52]. For example, Aβ and tau were detected in the exosomes of Alzheimer's Disease (AD) patients, and studies have shown disease propagation with exosomes containing Aβ42 oligomers [53, 54, 55]. Today, exosomes are used to early detect AD. For example, when GAP43, neurogranin, synaptotagmins, Rab3A, and SNAP25 are present in the exosomes of AD patients, this helps detect preclinical AD about 5–7 years prior to symptom onset [56, 57].

Understanding the role of exosomes in ALS is of great importance [58, 59, 60]. Diseased cells with SOD1 mutations propagated the mutated protein via exosomes [61], and SOD1^G93A^ mouse models had higher amounts of mSOD1 in their exosomes at later stages when compared to healthy controls [62]. Similarly, exosomes isolated from plasma [63] or post‐mortem brain samples [64] of ALS patients had increased levels of TDP‐43 protein. Recent studies detected FUS, C9orf72, and VCP in the extracellular vesicles isolated from ALS patients [65, 66, 67], suggesting a significant benefit for understanding the content of exosomes, with respect to disease progression. The plasma circulating extracellular vesicles were screened by RT‐qPCR and microarray approaches, especially to detect microRNA [68]. Initial proteomic studies of exosomes isolated from the CSF of ALS patients began to reveal how informative the protein content of exosomes is, and that one can learn about the disease state by studying the exosome proteins [69]. Therefore, we performed an unbiased discovery mode proteomics to reveal the content of exosomes isolated from the circulating serum of ALS patients with SOD1^D90A^ mutations.

One of the strengths of this study is its ability to study members of the same family at different stages of the disease. The Daughter is at the very early asymptomatic stage, the Son just becomes symptomatic, and the Father is symptomatic. In addition, having access to the serum of Patient #2, who has the same mutation but is even further progressed in his disease stage, allows longitudinal time points for investigations.

In large cohort studies, it is possible to have adequate numbers of age‐and sex‐matched controls, allowing proper power analyses. However, in our study, due to small sample size and intrinsic differences among control samples, performing comparisons within two time points of the same patient was found to be more reliable and informative. We, therefore, compared findings from T2 and T1 time points of the Daughter and the T2 and T1 time points of the Son. The changes that happened within a year in the same person were of great importance in revealing the cellular changes that occurred in their body. Such intrinsic comparisons were not possible with an external healthy control.

We found striking differences between the Daughter and the Son. This could be due to incomplete penetrance of the disease and that some patients do not develop disease symptoms even though they have the mutation. The data obtained from the Daughter via the assessment of canonical pathways, upstream regulators, and protein network analyses suggested that the main problem was at the site of vasculature and that there were multifactorial problems with BBB and its stability. This is in line with previous findings showing that the BBB defects occur prior to symptom onset [70]. Phosphorylation of proteins, increase in DNA repair machinery, mitochondrial defects, and initiation of the innate immunity also appear to take place prior to symptom onset. Albeit she remained asymptomatic, her body was actively trying to cope with the disease‐related damages and was beginning to show early signs at the cellular level, which have not yet been translated to clinical outcomes.

The Son, as he became symptomatic, displayed a very different profile than the daughter. For example, the DNA repair was not relevant for him. His body activated many of the cellular events related to neuroimmune modulation. The TRIM21 signaling pathway was activated, suggesting defects in his mitochondria triggered a mitochondria‐mediated immune response [36, 71]. Cellular infiltration to the CNS was occurring, the communication between innate immunity and adaptive immunity was taking place, and cellular events that lead to astrogliosis and microgliosis were present.

When Father and T2 time point of the Son were compared, the difference was striking. Pathways that are related to organismal death and phagocytosis were now upregulated. Tissue damage and gliosis took center stage, and none of the cellular pathways that are important for maintaining homeostasis were highlighted. At this stage, which is usually when most patients receive diagnosis, there was severe destruction and activation of the complement cascade. These findings further emphasize the importance of early diagnosis and early intervention.

We next investigated for the presence of proteins that display either increasing or decreasing profiles with respect to disease progression. FN1 protein levels were linearly increasing with respect to disease progression and decreasing ALSFRS scores.

FN1 is a glycoprotein present mostly in the extracellular matrix. It is mostly involved in cellular migration, regulation of blood coagulation, and the host defense system [72]. The FN1 has been previously associated with ALS. For example, proteomic studies using the spinal cord of SOD1^G93A^ mice at an early stage revealed high levels of FN1 protein [73]. The proteomic analyses of motor neurons isolated from ALS patients and controls also revealed the presence of high levels of FN1 in the patient population [74]. We found FN1 to be one of the key proteins in ALS and HSP/PLS proteome network, identified by the common binding partners of UMN disease‐related proteins [75]. The integrity of the BBB depends on collagen type IV, FN1, and laminin. Therefore, FN1 plays a significant role in maintaining the stability of the BBB. Our studies suggesting problems with the BBB early in the disease and showing a linear increase in the levels of FN1 in the exosomes of patients' serum implicate the importance of maintaining the stability of the BBB in ALS.

Since SOD1^D90A^ mutation is mostly observed in ALS patients with prominent UMN loss, it could be possible that changes in the levels of FN1 may offer an insight into the timing and the extent of UMN loss in patients. It is important to further investigate whether FN1 levels would change with disease progression in a larger cohort and whether it would respond to drug treatment, serving as a pharmacokinetic biomarker.

Bioinformatics has an essential role in the analysis and interpretation of proteomic data. The choice of protein database is crucial for the success of proteomic analyses. Filtering each dataset by target‐decoy with reversed protein sequences is important, as the inclusion of many weak or suspect protein identifications may lead to misinterpretation of the data. We applied the most stringent parameters in our investigations, and our finding is confirmed with ELISA studies. Our study shines light on the dynamic changes that occur at the cellular and network level with respect to disease progression and suggests FN1 as a potential pharmacokinetic biomarker for ALS patients, especially with prominent UMN involvement.

## Author Contributions

M.G. performed exosome isolation, proteomic analyses, and helped with the writing of the manuscript; A.G. obtained IRB approval and prepared consent forms; H.I. recruited patients and isolated serum; M.Y. and N.B. performed genomic analyses and determined the mutation; P.H.O. conceptualized and organized the study, analyzed data together with H.I., and wrote the manuscript.

## Conflicts of Interest

The authors declare no conflicts of interest.

## Supporting information


**File S1:** Detailed protocol for mass spectrometry‐based proteomics.

**Table S1:** The comparative list of proteins that are detected in the exosome of serum isolated from the daughter at T1 and T2, and that either show an increasing or decreasing profile.

**Table S2:** The comparative list of proteins that are detected in the exosome of serum isolated from the son at T1 and T2, and that either show an increasing or decreasing profile.

**Table S3:** The comparative list of proteins that are detected in the exosome of serum isolated from father and the T2 time point of the son, and that either show an increasing or decreasing profile.

## Data Availability

The data that supports the findings of this study is available in the Supporting Information of this article.
