# Supplementary material for: Exosome Proteomics of SOD1D90A Mutation Suggest Early Disease Mechanisms, and FN1 as a Biomarker
Source: Ann Clin Transl Neurol. 2025 Sep 29;13(1):131–43. doi: 10.1002/acn3.70208 (PMC12790174; doi:10.1002/acn3.70208)
Supplement: Supplementary file 1 — File S1: Detailed protocol for mass spectrometry‐based proteomics. [file ACN3-13-131-s002.docx]

**Supplementary file: Detailed protocol for mass spectrometry-based proteomics.**

Precipitated protein pellets were solubilized in 100μl of 8M urea for 30 min and then 100μl of 0.2% ProteaseMAX (Promega) was added for 2h. Protein extracts were reduced and alkylated as previously described^1^, followed by the addition of 300μl of 50mM ammonium bicarbonate, 5μl 1% ProteaseMAX and 0.5μg sequence-grade trypsin (Promega). Samples were digested overnight in a 37°C thermomixer (Eppendorf). For Orbitrap Fusion Tribrid MS analysis, the tryptic peptides were purified with Pierce C18 spin columns (Thermo Scientific). 3mg of peptide was auto-sampler loaded with a Thermo EASY nLC 1000 UPLC pump onto a vented Acclaim Pepmap 100, 75μm × 2cm, nanoViper trap column coupled to a nanoViper analytical column (Thermo-164570, 3μm, 100Å, C18, 0.075mm, 500mm) with stainless steel emitter tip assembled on the Nanospray Flex Ion Source with a spray voltage of 2,000V. Buffer A contained 94.785% H2O with 5% acetonitrile and 0.125% formic acid, and buffer B contained 99.875% acetonitrile with 0.125% formic acid. The chromatographic run was for 4h in total with the following profile: 0–7% over 7min, ramp to 10% over 6min, ramp to 25% over 160min, ramp to 33% over 40min, ramp to 50% over 7min, ramp to 95% for 5min and stay at 95% for 15mins. Additional MS parameters include: ion transfer tube temperature=300°C, Easy-IC internal mass calibration, the default charge state was set to 2s and cycle time was set to 3s. Detector type set to Orbitrap, with 60,000 resolution, with wide quad isolation, mass range was set to normal, scan range was set to 300–1,500 (m/z), max injection time was set to 50ms, AGC target was set to 200,000, microscans was set to 1, S-lens RF level was set to 60, without source fragmentation, and data type was set to positive and centroid.

Monoisotopic precursor selection is set as on, included charge states equal to 2–6 (and reject unassigned). Dynamic exclusion enabled and set to 1 for 30s and 45s exclusion duration at 10p.p.m. for high and low. Precursor selection decision is set to most intense, top 20, isolation window was set to 1.6, scan range was set to auto normal, first mass was set to 110, collision energy was set to 30%. For CID, we used the ion trap detector, ion trap resolution was set to 30 K, ion trap scan rate is set to rapid, maximum injection time was set to 75ms, AGC target was set to 10,000, and Q was set to 0.25, finally we injected ions for all available parallelizable time. Spectrum raw files were extracted into ms1 and ms2 files using the in-house program RawXtractor or RawConverter (<http://fields.scripps.edu/downloads.php>), and the tandem mass spectra were searched against UniProt human database (The UniProt Consortium 2015) and matched to sequences using the ProLuCID databse search program (ProLuCID ver. 3.1). ProLuCID searches are done on an Intel Xeon cluster running under the Linux operating system. The search space included all fully and half-tryptic peptide candidates that fell within the mass tolerance window with no miscleavage constraint. The validity of peptide/spectrum matches (PSMs) is assessed in DTASelect2 (using two SEQUEST-defined parameters, the cross-correlation score (XCorr), and normalized difference in cross-correlation scores (DeltaCN)^2-4^. The search results are grouped by charge state (+1, +2, +3, and >+3) and tryptic status (fully tryptic, half-tryptic, and nontryptic), resulting in 12 distinct subgroups. In each of these subgroups, the distribution of Xcorr, DeltaCN, and DeltaMass values for (1) direct and (2) decoy database PSMs are obtained. Peptide match probabilities are calculated based on a nonparametric fit of the direct and decoy score distributions.

**References**

1. Muller T, Winter D. Systematic Evaluation of Protein Reduction and Alkylation Reveals Massive Unspecific Side Effects by Iodine-containing Reagents. Mol Cell Proteomics. 2017 Jul;16(7):1173-87.

2. Klammer AA, Park CY, Noble WS. Statistical calibration of the SEQUEST XCorr function. J Proteome Res. 2009 Apr;8(4):2106-13.

3. Tabb DL, McDonald WH, Yates JR, 3rd. DTASelect and Contrast: tools for assembling and comparing protein identifications from shotgun proteomics. J Proteome Res. 2002 Jan-Feb;1(1):21-6.

4. Eng JK, Searle BC, Clauser KR, Tabb DL. A face in the crowd: recognizing peptides through database search. Mol Cell Proteomics. 2011 Nov;10(11):R111 009522.
